# Job Quality in the Late Career in Sweden, Japan and the United States

**DOI:** 10.1177/01640275221075985

**Published:** 2022-05-19

**Authors:** Loretta G. Platts, Lawrence B. Sacco, Ayako Hiyoshi, Hugo Westerlund, Kevin E. Cahill, Stefanie König

**Affiliations:** 1 Department of Psychology, Stress Research Institute, 123910 Stockholm University, Stockholm, Sweden; 2 Clinical Epidemiology and Biostatistics, School of Medical Sciences, 6233 Örebro University, Örebro, Sweden; 3 6019 Center on Aging & Work at Boston College, Boston, MA, United States; 4 Department of Psychology, and Centre for Ageing and Health, 3570 University of Gothenburg, Gothenburg, Sweden

**Keywords:** post-retirement work, working conditions, working retirees, health and retirement study, international comparative study

## Abstract

Increasing numbers of older workers continue to work after being eligible to claim a state pension, yet little is known about the quality of these jobs. We examine how psychosocial and physical job quality as well as job satisfaction vary over the late career in three contrasting national settings: Sweden, Japan and the United States. Analyses using random effects modelling drew on data from the Swedish Longitudinal Occupational Survey of Health (*n* = 13,936–15,520), Japanese Study of Ageing and Retirement (*n* = 3704) and the Health and Retirement Study (*n* = 6239 and 8002). Age was modelled with spline functions in which two knots were placed at ages indicating eligibility for pensions claiming or mandatory retirement. In each country, post-pensionable-age jobs were generally less stressful, freer and more satisfying than jobs held by younger workers, results that held irrespective of gender or education level.

## Introduction

A well-established practice worldwide is to continue in paid work after becoming eligible for old-age state pensions. Among Organisation for Economic Co-operation and Development (OECD) member countries, for example, about 20% of 65–69 year olds are in paid work (OECD, 2017, pp. 124–126). High exit ages are often viewed favourably by policymakers, as continued work later in life alleviates some of the financial strains of an ageing society. Underlying this perspective, however, is an assumption that these jobs are not characterized by a reduction in job quality and, concomitantly, in the well-being of older individuals. Somewhat surprisingly, little is known about the quality of post-pensionable-age jobs, and whether older adults are segregated into poor quality work.

Much research into the late career focusses on jobs held prior to pensionable age, while studies that do extend into older ages usually examine whether people work at all (McAllister et al., 2019). While some recent studies have examined the nature of jobs held over pensionable age (Brussig, 2016; Wahrendorf et al., 2017), little research has examined how job quality relates to dimensions of disadvantage such as gender or socio-economic position (exceptions are Dingemans & Henkens, 2020; Weber et al., 2018). Further, a recurring theme in the late career literature is the question of whether lack of social protection may compel older adults to remain in work, with the implication that job quality may be poorer in less protective countries. However, internationally comparative research examining job quality after pensionable age is lacking (Dingemans & Henkens, 2020).

We extend the literature by examining job quality over a wide span of the late career, including jobs held after normal full-pension-eligibility ages in three countries. We examine whether older workers in general and workers over pension age in particular tend to be segregated into poor quality jobs and whether certain groups – women and people with fewer formal qualifications – are at particular risk of being in poor quality jobs at these ages. The national context may be consequential for job quality in the late career (Scherger, 2015). This macro-level perspective is attended to by comparing three countries – Sweden, the US and Japan – that have attained high employment rates among older workers through contrasting institutional arrangements. We seek to illustrate communalities and differences in the nature of jobs available to post-pensionable-age workers in mature welfare states.

## Theoretical Framework

### Might There Be an Age–Job Quality Relationship?

The literature examining the nature of job quality in the late career is small, and we consequently incorporate wider factors to inform our hypotheses concerning the potential relationship between age and job quality in this life stage. Our theoretical starting point is that the relationship between job quality and age in the late career is determined simultaneously by how older workers fare against others in market-based job allocation processes and by normative and financial pressures to work.

We first present a line of reasoning that would anticipate lower job quality in the latter part of the late career. Compared with core-age workers, older workers typically have a position of disadvantage on the labour market: they may be subject to age discrimination, may not have been offered sufficient training to keep their skills up-to-date and, if they lose their job, risk being unemployed for a long time (Dingemans et al., 2016; Hardy, 1991; Sjöström & Örnhall Ljungh, 2011). Lowered demand for a person’s labour will result in the person falling back in the labour queue to less appealing, poorer quality jobs (Taylor & Walker, 1994). While older workers may cease their participation in the labour market once the quality of available jobs drops too low, individuals experiencing strong normative and financial pressures to work may have to take such jobs. Empirical support for lower job quality in the late career is provided by Lain (2012) who observed that, compared to employees aged 58–59 years, British employees older than 65 were disproportionately employed in low paid occupations requiring few formal qualifications.

Another line of reasoning leads to the opposite conclusion: the quality of jobs held by older workers will be high. With children grown up and access to pensions no longer a distant prospect, older adults may experience fewer financial and normative pressures to work compared to younger age groups for two reasons. Firstly, decommodified to some degree from the labour market, older workers may be able to refuse poor quality work and retire instead (Smeaton et al., 2009, p. 17). Differential selection of workers with poorer working conditions into retirement has been demonstrated in the United Kingdom; this would raise average quality of late career jobs as cohorts aged (Carr et al., 2016; Smeaton et al., 2009, p. 17). Secondly, older adults may be able to withstand periods out of the labour market to find a better job, which may enhance their leverage vis-à-vis employers in crafting jobs that suit themselves better (Kooij et al., 2020; Wrzesniewski & Dutton, 2001). The little empirical evidence that exists suggests that the quality of jobs held by workers in their sixties and seventies might typically be better than jobs held by workers in their fifties. In Europe, working retirees and workers over pensionable age reported more favourable physical and psychosocial working conditions than younger workers, specifically lower physical demands and effort reward imbalance and higher control (Brussig, 2016; Dingemans & Henkens, 2020; Topa et al., 2014; Wahrendorf et al., 2017). Based on these observations, we ask: *Might there be an age–job quality relationship during the late career?* Because strong arguments can be made in both directions, we propose the following opposing hypotheses:

Hypothesis 1a Within the late career, job quality tends to be lower at older ages.

Hypothesis 1b Within the late career, job quality tends to be higher at older ages.

### Might There Be an Impact on Job Quality of Having Attained Pensionable Age?

The above-mentioned processes may operate gradually, generating a smooth age–job quality relationship. However, cultural schema and institutional clocks are organized in relation to certain chronological ages, generating step changes in the life course (Moen, 2011). Break-points in how far older adults are decommodified from the labour market may occur as adults exceed ages that are the criteria for access to old-age pensions and, in some settings, mandatory retirement ages and improved healthcare coverage (e.g. Medicare in the United States available to most citizens from 65 years). An additional factor is economic incentives in the form of reduced social security and tax payments offered by many governments to employers and employees that reduce the cost and increase the returns of hiring workers older than pensionable age.

Such age-graded policies, practices and cultural beliefs may generate abrupt contrasts in job quality before and after certain key ages. These may occur in two contrasting directions. In terms of anticipated declines in job quality, once eligible for old-age pensions, older adults may encounter heightened social expectations to retire from the labour market. From this perspective, we might expect that attaining an age of pension eligibility may be associated with a break-point in the age-job quality relationship such that older workers report poorer job quality. Again, the converse can be argued: the jobs held by the oldest workers might be of better quality than those held by younger workers since financial security is offered by old-age pension receipt and, further, national policies often set lower employer charges and tax rates for workers over pensionable age, factors which boost their desirability to employers. Employers may correspondingly be incentivized to improve aspects of working conditions in order to retain their oldest employees (Kohli & Rein, 1991, p. 18). These mechanisms would generate raised average quality of jobs held after the break-point of attaining pensionable age.

Consequently, we ask: *Might there be an impact on job quality of having attained pensionable age?* and propose the following opposing hypotheses:

Hypothesis 2a Pensionable ages mark a break-point in the age–job quality relationship.

Hypothesis 2b Pensionable ages do not mark a break-point in the age–job quality relationship.

### Individual-level factors stratifying access to good quality work in the late career

It is well established that processes of selection into paid work favour certain individual characteristics and circumstances, leading to inequalities in access to late career jobs (Hasselhorn, 2020; Platts et al., 2019; Pleau, 2010), and potentially also inequalities in job quality. Women and low-skilled workers, as a result of lower lifetime earnings, are likely to embark on the late career from a position of fewer resources and may be more likely to prolong their working lives for financial reasons (Hofäcker & Naumann, 2015; Street & Léime, 2020). The same groups typically find themselves further towards the back of the labour queue (McAllister et al., 2019): Employment rates for women tend to be lower than those for men, both generally and later in life (Coile, 2020; Laun & Palme, 2020; Oshio et al., 2020) and people with few skills may be competing for jobs against more skilled workers prepared to occupationally downgrade. Consequently, women and low-skilled workers might be less likely to have good jobs in the late career. We ask: *Are there differences in the quality of the jobs held in the late career by gender and education level?* and hypothesize that:

**Hypothesis 3:** The quality of jobs held in the late career is poorer for women than for men.
**Hypothesis 4:** The quality of jobs held in the late career is poorer for people with a lower education level compared to people with a higher education level.

Further, attaining the age of old-age pension eligibility may make less difference for women’s job quality than for men. This is because, in all three countries we examine, women over 65 years are more likely to be living in relative poverty than men (OECD, 2019b, p. 187) and would consequently benefit more by supplementing their pension income with labour earnings (Street & Léime, 2020). Therefore, women may be more likely than men to accept remaining in poor quality jobs even after pensionable age. Only two studies have examined gender differences in the development of working conditions by age: Dingemans and Henkens (2020) found no differences by gender; Brussig (2016) observed limited differences.

Similarly, attaining the age of old-age pension eligibility may make less difference for job quality of people with fewer formal qualifications than it does for people with more. Improving job satisfaction and psychological working conditions through downward occupational mobility and job changes may be more difficult for low-skilled workers (Nekola et al., 2018; Sacco et al., 2021). Therefore, we seek to bolster the limited existing evidence by asking: *Are there differences by gender or by education level in the association between age and working conditions?* and hypothesize that:

Hypothesis 5 The effects of attaining pensionable age on the age–job quality relationship are smaller for women than for men.

Hypothesis 6 The effects of attaining pensionable age on the age–job quality relationship are smaller for people with a lower education level compared to people with a higher education level.

### Comparing Three Persistent Late Exit Countries: Sweden, Japan and the United States

We incorporate a macro-level perspective on the late career (Hess et al., 2016) because cultural norms, social welfare systems and labour markets all shape the institution of retirement and, by extension, work in later life (Szinovacz, 2013). Such institutions and norms affect work opportunities (e.g. via mandatory retirement ages) as well as the financial pressures to work older adults face (e.g. via pension replacement rates). Cross-nationally, the degree to which welfare arrangements in general and old-age pensions in particular decommodify older adults from the labour market may be a factor determining how likely older adults are to retire from poor quality jobs. In this vein, a cross-comparative study of European countries observed poorer job quality among 60–75-year-olds in settings with higher levels of material deprivation (Dingemans & Henkens, 2020). Little is known about how attaining age of state pension eligibility might shape job quality, in particular outside of Europe. Consequently, we extend prior cross-national research by comparing the European case of Sweden with two non-European countries: Japan and the United States.

These countries, drawn from three continents, share substantial similarities. High employment rates among older workers led to their classification as ‘persistent late exit’ countries (Hess et al., 2016; OECD, n.d.; Table 1). Each offers financial incentives for delaying pension claiming and working after pensionable age: labour earnings generally exceed old-age pensions and delays to claiming state pensions lead to actuarial increases in payments (Haaga & Johnson, 2012). However, re-employment opportunities for older people are fewer than for core-age workers; non-standard employment (e.g. shortened working time, short-term contracts) and reduced wages are more common at older ages as well (Higo et al., 2016; OECD, 2020b). Concerning pension generosity, net replacement rates from mandatory pensions were in each country under the OECD average (Table 1). In each country, while lower earners are protected to some degree – net replacement rates are higher for those on half average compared to average earnings – inequalities remain: relative poverty rates for people over 65 are higher than for core-age adults (OECD, 2019a, p. 101) and more women over 65 live in relative poverty than men (OECD, 2017, p. 109; Table 1).

**Table 1.** Comparison of three persistent late exit countries: Sweden, Japan and the United States.

Characteristic	Sweden	Japan	United States
Labour force participation rates (2015, %) (1)			
65–69 years	22%	43%	32%
70–74 years	10%	25%	19%
Age-based mandatory retirement (2015)	At 67 years	At 60 years	Age-discrimination legislation prohibits mandatory retirement
Pensions			
Net replacement rates from mandatory pensions (% of prior earnings) (2)	55%	40%	49%
Net replacement rates from mandatory pensions and voluntary contributions (% of prior earnings) (2)	55%	64%	87%
Net replacement rates from mandatory pensions and voluntary contributions (% of prior earnings) for lower earners (earning 50% of average wage) (2)	62%	79%	97%
Relative poverty rates (%)			
Total population (3)	9%	16%	18%
People aged 66–75 years (3)	8%	17%	20%
People aged >75 years (3)	17%	23%	28%
Men >65 years (3)	7%	16%	20%
Women >65 years (3)	15%	22%	26%
Age thresholds (2015)			
Age 60		Mandatory retirement age	
Age 61	Earliest age to claim state old-age pension		
Age 62			Earliest age to claim Social Security benefits
Age 65	Eligibility ceased for unemployment benefits; eligibility more restrictive for sickness benefits; eligibility began for minimum income guarantee old-age pension	Old-age pensions usually begin to be paid from this age	Medicare benefits become available
Age 66			Cohorts born before 1955 can access unreduced Social Security retirement benefits
Age 67	Legal employment protection ceased		

Sources: (1) (OECD, 2020a), (2) (OECD, 2017, p. 109), (3) Relative poverty rate is percentage with income lower than 50% of median equivalized disposable income, 2016 or latest available year (OECD, 2019b, p. 187).

Alongside these similarities are contrasts in cultural expectations and welfare state arrangements for older people. Sweden provides a safety net for poorer pensioners – poverty rates for over 65s are substantially lower in Sweden than in the other two countries – but few employment rights (OECD, 2019b, p. 187; Table 1). Despite some limited incentives for hiring post-pensionable-age workers – employee taxes and employer social security charges drop substantially after 65 – mandatory retirement at age 67 is a general practice (Zettervall, 2013) and workers past that age are no longer protected by labour law. While it is likely more difficult to remain in work at post-pensionable ages (after 67 years) in Sweden than in the other two countries, the social protection provided by pensions would facilitate refusing poor quality jobs.

In the United States, access to benefits for those with low pensions is limited in a model of ‘self-reliance’ (Lain, 2016). Civil rights, in contrast, are strong: age-discrimination legislation prohibits age-based mandatory retirement in the United States. Further, limits on earnings that can be received whilst receiving retirement benefits from Social Security are removed once beneficiaries reach full retirement age, providing an incentive to work (Haaga & Johnson, 2012). While it is probably easier to remain in work after pensionable age in the United States than in Sweden or Japan, relatively high rates of pensioner poverty might drive participation in poor quality jobs.

Japan’s Confucian culture, with its strong work ethic and respect for older people’s experiences and knowledge, has historically placed a strong value on remaining in paid work in old age (Debroux, 2016). However, there is evidence of underemployment of older Japanese as a result of limited employment opportunities (Usui et al., 2016). Further, while people could access their old-age pension from 65 years during the period of our study from the late 2000s to the early 2010s, mandatory and common retirement ages were only 60 years (Takayama, 2013). This long-standing gap between retirement age and pensionable age has led to the custom of people working in public sector employers or large companies who turn 60 ‘descending’ to a lower position: working in an affiliated company or being re-employed with reduced wage and responsibilities (Clark & Ogawa, 1997; Higo et al., 2016); other employees may lack these options. To conclude, financial pressures to remain in the labour market following an initial mandatory retirement might encourage take-up of poor quality jobs in the period between retirement and pensionable age.

In short, these countries have attained high rates of labour participation in later life by contrasting paths. With more protective pensions in Sweden compared to the United States or Japan, financial need may be of lesser importance in causing people to stay in the labour market, and Swedes may remain in work only if job quality is sufficiently good. In addition to this selection mechanism, job quality may improve if workers, supported by the financial cushion of a pension, are able to alter aspects of their jobs by negotiating with employers to improve their working conditions. The relative strength of these two mechanisms in Sweden might generate larger impacts of attaining pensionable age upon perceived job quality in Sweden compared to Japan or the United States. We consequently ask: *Are there differences by macro-setting in the development of working conditions by age?* and hypothesize that:

Hypothesis 7 The effects of attaining pensionable age on the age–job quality relationship may be larger in Sweden than in the United States or Japan.

### Delimiting Pensionable Ages in Sweden, Japan and the United States

In each country, a range of threshold ages demarcate access to state old-age pensions, employment protection and out-of-work benefits (Table 1). We conceptualized these ages as delimiting three periods: pre-pensionable ages, pensionable ages and post-pensionable ages. Reforms of pension ages have been implemented from 2020 in Sweden and Japan; consequently, we describe threshold ages in Sweden and Japan as they were during the data collection period lasting from 2006 until 2018 (Sweden) and until 2013 (Japan).

Prior to 2020, Sweden had a flexible state pension age between 61 and 67 years, with the age of old-age pension eligibility preceding the mandatory retirement age. Age 61 delimits the start of pensionable years, albeit with reductions for claiming early, and age 67, when legal employment protection ceased, delimits the start of the post-pensionable years (König & Sjögren Lindquist, 2016). In Japan, in contrast, the mandatory retirement age precedes the age of eligibility for the state old-age pension. For the period under study, pensionable age in Japan began at age 60, corresponding with mandatory retirement; the start of post-pensionable age is age 65, when old-age pensions are usually paid (Ohashi, 2008). We set the beginning of the pensionable years in the United States at age 62, when it becomes possible to access Social Security benefits, albeit with reductions for claiming early. Cohorts born before 1955 can access unreduced Social Security retirement benefits from 66 years, marking the start of the post-pensionable years (Social Security Administration, 2020).

### Measuring Work Quality

We attend to diverse aspects of the multidimensional concept of work quality because different aspects of job quality may change in distinctive ways as workers approach and exceed pensionable age. Some aspects of work quality might be more elastic than others; alternatively, workers may trade off some aspects of work quality for others. For example, late career shifts into manual work can lead to reduced workload pressure but increased physical demands (Nekola et al., 2018). Further, the relative importance of various aspects of working conditions may change during the late career. Once eligible for an old-age pension, any declines in earnings and job security may matter less while more intrinsic aspects of the work environment may matter more (Platts et al., 2021). We consequently include a measure of job satisfaction to observe overall perceived job quality.

We draw on two complementary theoretical approaches to measuring job quality: the demand-control model – focussing on job tasks – and the effort reward imbalance model – focussing on labour market aspects. In the demand-control model, strain results from the relationship between the demands of the work situation and the decision-making freedom (discretion) available to the worker (Karasek, 1979). The effort reward imbalance model examines social reciprocity in the job contract, in which employee efforts should be matched by the rewards (earnings, promotion or job security, and esteem) provided by the employer (Siegrist, 2017).

## The Present Study

We seek with this paper to present new evidence about psychosocial and physical working conditions and job satisfaction in the late career, using panel data drawn from community-based samples in Japan, Sweden and the United States and harmonized *ex post*. In addition to analyzing the age–job quality relationship, we examine whether there might be an impact on job quality of having attained pensionable age. Lastly, we seek to observe heterogeneity in these relationships, in relation to gender, education level and country.

## Research Design

### Data

Data were drawn from three national biennial surveys of ageing harmonized *ex post*. Swedish data came from the biennial Swedish Longitudinal Occupational Survey of Health (SLOSH, 2006–2018, *N* = 40 877) (Magnusson Hanson et al., 2018). The survey follows up respondents from the Swedish Work Environment Survey 2003–2011, a sample drawn from the Swedish working population aged 16–64 years. Data collected by postal questionnaires were linked to administrative registers, for example, the longitudinal integrated database for health insurance and labour market studies (LISA). Participants in paid work for at least 30% of full time were asked to complete an ‘in-work questionnaire’, others a ‘non-worker questionnaire’. See Supplementary Table A1 and Supplementary Figure A1 for a description of which working conditions were measured in the two questionnaires at each survey wave. Stockholm’s Regional Research Ethics Board has approved SLOSH.

The Japanese Study of Ageing and Retirement (JSTAR, 2006–2013, *N* = 15 500) is a face-to-face interview study of people aged over 50 years from 10 prefectures which is designed to be comparable with other ageing surveys. Respondents from five cities were originally surveyed in 2006; two prefectures were added in the 2009 follow-up and three more added in 2011 (RIETI, 2016).

The Health and Retirement Study (HRS) is a longitudinal study of older individuals living in the United States. The study began in 1992 by interviewing individuals aged 51–61 and their spouses face-to-face and has since been supplemented with additional cohorts of older Americans. Follow-up interviews are conducted every second year and over 37 000 Americans have been surveyed (Sonnega et al., 2014). Data on working conditions were available in waves 8–11 (2006–2012) and for job satisfaction additionally in waves 12 and 13 (2014 and 2016); participants responded to these modules at alternate waves (4-year interval).

The final sample of respondents for this paper were selected by retaining records at any wave where participants, aged between 50 and 75 years, were currently in paid work for one or more hours weekly. Records were excluded if they lacked information on age or gender. In Sweden, the survey instrument changed over time (cf. Supplementary Figure A1 in appendix) and sample sizes consequently differed somewhat across the outcomes from 13,931 to 15,512 participants. Since job quality measures were collected at all waves in the Japanese data, records lacking information for any job quality item were excluded, generating a national sample of 3690 participants. There were two American samples, one containing 7952 participants for the job satisfaction outcome and 6198 participants for the other job quality outcomes.

### Variables

Full details about the harmonized variables in each dataset are provided in Appendix (Supplementary Table A1).

#### Demographics

Demographic information was obtained from administrative registers in SLOSH and from self-reports in JSTAR and HRS. Age was centred on 50 years and gender coded as 0 = male, 1 = female. Education level obtained from self-reports was recoded according to the International Standard Classification of Education (ISCED) 1997 into the categories: up to lower secondary (ISCED 0–2), upper secondary (ISCED 3–4) and tertiary (ISCED 5–6).

#### Perceived Job Quality and Job Satisfaction

Perceived job quality and job satisfaction were measured with six single-item questions in each survey; full details are provided in Supplementary Table A1. The items were physical labour (e.g. ‘My job requires lots of physical effort’), time pressure (‘I have constant time pressure due to a heavy workload’), discretion (e.g. ‘I have very little freedom to decide how I do my work’), satisfaction with pay (e.g. ‘My income is adequate’), job security (‘My job security is poor’) and job satisfaction (e.g. ‘Overall, I am satisfied with my current job’). Nearly all items were originally measured on a 1–4 scale; items with longer scales were converted to this range. Items were reversed-scored if necessary so that higher scores represent higher values of the construct (e.g. greater discretion). Time pressure due to heavy workload corresponds to the demand dimension of the demand-control model and to the efforts dimension of the effort reward imbalance model. Physical labour also corresponds to efforts for jobs involving such labour. Satisfaction with pay and job security stem from the reward dimension of the effort reward imbalance model. Discretion stems from the decision-authority subscale of the control dimension of the demand-control model. We calculated effort reward imbalance as follows:

$$Effort\;reward\;imbalance=\frac{time\;pressure\;from\;heavy\;workload}{0.5\times \left(pay+job\;security\right)}$$

### Analysis

Stockholm’s Regional Research Ethics Board approved the present study (2019–01637). Analyses were performed in Stata SE 16.1. Descriptive statistics were used to summarize the sample characteristics for the sample as a whole and by age group at wave of entry. We modelled the panel data using random intercept models with maximum likelihood estimation, an approach nesting observations within individuals to estimate a mixture of between and within-unit variance of the dependent variable (Andreß et al., 2013). The use of person-specific random intercepts assumes an exchangeable correlation structure, in which the amount of correlation is the same between each measurement. Because the models incorporate between-individual variation in addition to within-individual variation, the results should not be interpreted as estimates of within-individual changes in job quality.

We used two specifications for age – linear and spline functions – and used the likelihood ratio test to examine which was the best fitting function. Our first set of models are the simplest, using a linear age specification. If these are the optimal models, it would provide an indication that the relationship between job quality and age is a gradual one, merely reflecting linear age differences. Our second set of models incorporated knots at ages which marked ages of pension eligibility and changes to labour market rules for older people (Sweden: 61 and 67 years; Japan: 60 and 65 years; United States: 62 and 66 years). Using knots at these pre-defined age boundaries offers the advantages of being relatively parsimonious and of being a theoretically-led approach. If spline modelling offers better fit than the linear functions for the job quality-age relationship, this would indicate that there are changes in slope in this relationship at one or both of the knots delimiting the three periods that reflect institutional age thresholds. In the spline models, beta coefficients indicate slopes for each spline; margins show changes in slope from one spline to the next. Figures are presented with spline functions, since this is the most flexible specification.

We performed two sets of analyses to examine relationships with gender and education level: (1) including gender/education level as a covariate in order to observe whether mean levels of a given outcome over the full age range from 50 to 75 years varied by gender or education level, and (2) introducing interactions between gender/education level and the linear term/spline terms, as appropriate, in order to observe whether the gradients of the age slopes varied differently by gender or education level.

We performed sensitivity analyses with random-effects ordered logistic models as a robustness check given that the job quality outcome measures were measured on a four-point scale.

## Results

### Descriptive Findings

In each country sample, men made up more than one-half of workers at post-pensionable ages. In Sweden, the proportion of workers who were men among the post-pensionable age sample was 23.9 percentage points higher than the proportion who were men among the pre-pensionable age sample (66.9% vs. 43.0%) (Table 2). In Japan, the proportion was 5.4 percentage points higher (61.5% vs. 56.1%), and in the United States, the proportion was 8.4 percentage points higher (51.3% vs. 42.9%). In Sweden, 39.1% of workers at pre-pensionable ages were educated to tertiary level, which increased to 50.4% of workers at post-pensionable ages. However, the education gradient was opposite in both Japan and the United States, such that the proportion of workers educated to tertiary level was lower at post-pensionable ages (14.3% in Japan and 47.8% in the United States) than at pre-pensionable ages (35.0% in Japan and 54.0% in the United States). Workers rated both the psychosocial work environment of their jobs and job satisfaction more positively at post-pensionable ages compared with pre-pensionable ages. However, there was little pre- and post-pensionable pattern across countries with respect to the physical labour-related items.

**Table 2.** Pooled descriptive statistics for Sweden (Swedish Longitudinal Occupational Survey of Health), Japan (Japanese Study of Ageing and Retirement) and the United States (Health and Retirement Study).

**Sweden**				
	Mean (sd) or *n* (%)			
Variable	All	Pre-pensionable ages: 50–60 years	Pensionable ages: 61–66 years	Post-pensionable ages: 67–75 years
Gender (physical labour sample, pooled *n* = 37719)				
Male	16730 (44.4%)	11765 (43.0%)	4451 (46.5%)	514 (66.9%)
Female	20989 (55.6%)	15624 (57.0%)	5111 (53.5%)	254 (33.1%)
Education level				
Up to lower secondary (ISCED 0–2)	6150 (16.3%)	4011 (14.6%)	2032 (21.3%)	107 (13.9%)
Upper secondary (ISCED 3–4)	16599 (44.0%)	12670 (46.3%)	3655 (38.2%)	274 (35.7%)
Tertiary (ISCED 5–6)	14970 (39.7%)	10708 (39.1%)	3875 (40.5%)	387 (50.4%)
*Total*	37719 (100.0%)	27389 (100.0%)	9562 (100.0%)	768 (100.0%)
Job quality (pooled *n*)				
Work physically (*n* = 37719)	3.17 (1.02)	3.15 (1.02)	3.21 (1.00)	3.35 (0.95)
Time pressure due to a heavy workload (*n* = 30671)	2.53 (0.86)	2.59 (0.85)	2.42 (0.86)	1.94 (0.88)
Discretion (*n* = 33570)	3.37 (0.76)	3.33 (0.77)	3.43 (0.75)	3.55 (0.70)
Pay satisfaction (*n* = 30671)	2.34 (0.89)	2.33 (0.88)	2.34 (0.90)	2.79 (0.93)
Job security (*n* = 30671)	3.56 (0.72)	3.53 (0.73)	3.64 (0.69)	3.71 (0.65)
Job satisfaction (*n* = 33818)	3.18 (0.64)	3.12 (0.64)	3.25 (0.63)	3.48 (0.57)
Effort reward imbalance (*n* = 30671)	0.92 (0.45)	0.94 (0.45)	0.87 (0.43)	0.64 (0.36)
**Japan (pooled *****n***** = 7026)**				
	Mean (sd) or *n* (%)			
Variable	All	Pre-pensionable ages: 50–59 years	Pensionable ages: 60–64 years	Post-pensionable ages: 65–75 years
Gender				
Male	4118 (58.6%)	1703 (56.1%)	1130 (59.5%)	1285 (61.5%)
Female	2908 (41.4%)	1332 (43.9%)	770 (40.5%)	806 (38.5%)
Education level (ISCED)				
Up to lower secondary (ISCED 0–2)	1477 (21.0%)	338 (11.1%)	378 (19.9%)	761 (36.4%)
Upper secondary (ISCED 3–4)	3733 (53.1%)	1636 (53.9%)	1067 (56.2%)	1030 (49.3%)
Tertiary (ISCED 5–6)	1816 (25.9%)	1061 (35.0%)	455 (23.9%)	300 (14.3%)
*Total*	7026 (100.0%)	3035 (100.0%)	1900 (100.0%)	2091 (100.0%)
Job quality				
Physical labour	2.31 (1.01)	2.31 (1.02)	2.32 (1.01)	2.29 (1.00)
Time pressure due to a heavy workload	2.28 (0.94)	2.49 (0.93)	2.22 (0.94)	2.04 (0.88)
Discretion	2.89 (1.05)	2.86 (1.02)	2.89 (1.07)	2.95 (1.08)
Pay satisfaction	2.52 (0.83)	2.46 (0.82)	2.50 (0.83)	2.63 (0.84)
Job security	3.16 (0.92)	3.12 (0.92)	3.16 (0.92)	3.22 (0.91)
Job satisfaction	2.97 (0.70)	2.90 (0.72)	2.98 (0.69)	3.07 (0.68)
Effort reward imbalance	0.87 (0.49)	0.97 (0.52)	0.84 (0.47)	0.75 (0.44)
**United States**				
	Mean (sd) or *n* (%)			
Variable	All	Pre-pensionable ages: 50–61 years	Pensionable ages: 62–65 years	Post-pensionable ages: 66–75 years
Gender (job satisfaction sample, pooled *n* = 11753)				
Male	5251 (44.7%)	3292 (42.9%)	833 (44.0%)	1126 (51.3%)
Female	6502 (55.3%)	4374 (57.1%)	1060 (56.0%)	1068 (48.7%)
Education level (ISCED)				
Up to lower secondary (ISCED 0–2)	685 (5.8%)	421 (5.5%)	110 (5.8%)	154 (7.0%)
Variable	All	Pre-pensionable ages: 50–61 years	Pensionable ages: 62–65 years	Post-pensionable ages: 66–75 years
Upper secondary (ISCED 3–4)	4883 (41.6%)	3108 (40.5%)	784 (41.4%)	991 (45.2%)
Tertiary (ISCED 5–6)	6185 (52.6%)	4137 (54.0%)	999 (52.8%)	1049 (47.8%)
*Total*	11753 (100.0%)	7666 (100.0%)	1893 (100.0%)	2194 (100.0%)
Job quality (pooled *n*)				
Physically demanding (*n* = 7735)	2.38 (0.99)	2.43 (1.01)	2.33 (0.97)	2.25 (0.95)
Time pressure due to a heavy workload (*n* = 7735)	2.11 (0.93)	2.22 (0.94)	2.06 (0.93)	1.78 (0.81)
Discretion (*n* = 7735)	3.21 (0.80)	3.15 (0.81)	3.30 (0.77)	3.35 (0.76)
Pay satisfaction (*n* = 7735)	2.74 (0.86)	2.68 (0.86)	2.81 (0.83)	2.88 (0.84)
Job security (*n* = 7735)	3.06 (0.86)	3.01 (0.87)	3.17 (0.80)	3.17 (0.84)
Job satisfaction (*n* = 11753)	3.34 (0.75)	3.27 (0.78)	3.41 (0.71)	3.50 (0.66)
Effort reward imbalance (*n* = 7735)	0.81 (0.53)	0.87 (0.56)	0.75 (0.47)	0.65 (0.42)

Note: ISCED: International Standard Classification of Education.

### Analytic findings

Main analyses were carried out with linear random effects modelling; sensitivity analyses were carried out with ordered logistic random effects models. Unless stated otherwise, the results from ordered logistic models were similar to the main findings.

Consistent with the descriptive findings, at older ages, psychosocial working conditions were better and job satisfaction was higher (Table 3: Linear age specification), a finding which runs counter to Hypothesis 1a: *Within the late career, job quality tends to be lower at older ages* and provides support for Hypothesis 1b: *Within the late career, job quality tends to be higher at older ages*. Differences by age in time pressure due to a heavy workload were particularly large. Also consistent with the descriptive findings, age differences in physical labour were inconsistent across countries. In Sweden, older workers reported more time working entirely physically, in Japan an age effect was not observable, and in the United States the oldest workers perceived their work as less physically demanding.

**Table 3.** Random effects modelling of working conditions and job satisfaction in Sweden (Swedish Longitudinal Occupational Survey of Health), Japan (Japanese Study of Ageing and Retirement) and the United States (Health and Retirement Study).

**Sweden (n=13931 to 15512)**															
	Age	Work physically		Time pressure		Discretion		Pay		Security		Job satisfaction		Effort reward imbalance	
*n*		15,512		13,931		14,718		13,931		13,931		14,741		13,931	
Sweden: Linear model															
Age	50–75 y	0.010***		−0.020***		0.008***		0.006***		0.014***		0.014***		−0.011***	
Constant		3.077***		2.666***		3.306***		2.289***		3.454***		3.058***		0.998***	
Sweden: Spline model															
Age categories		Coef.	Margins	Coef.	Margins	Coef.	Margins	Coef.	Margins	Coef.	Margins	Coef.	Margins	Coef.	Margins
Pre-pensionable ages	50–60 y	0.009***	—	−0.006***	—	0.001	—	−0.003	—	0.010***	—	0.002*	—	−0.005***	—
Pensionable ages	61–66 y	0.013***	0.004	−0.050***	−0.045***	0.023***	0.022***	0.027***	0.030***	0.027***	0.016***	0.040***	0.037***	−0.026***	−0.021***
Post-pensionable ages	67–75 y	0.004	−0.009	−0.076***	−0.026*	0.007	−0.016*	0.042***	0.014	−0.009	−0.035**	0.015***	−0.025***	−0.030***	−0.004
Constant		3.080***	—	2.608***	—	3.335***	—	2.326***	—	3.469***	—	3.108***	—	0.972***	—
LR test: spline versus linear		Linear (*p* = 0.440)		Spline (*p*<0.001)		Spline (*p*<0.001)		Spline (*p*<0.001)		Spline (*p*<0.001)		Spline (*p*<0.001)		Spline (*p*<0.001)	
**Japan (*****n***** = 3690)**															
	Age	Physical labour		Time pressure		Discretion		Pay		Security		Job satisfaction		Effort reward imbalance	
Japan: Linear model															
Age	50–75 y	0.002		−0.032***		0.007**		0.013***		0.010***		0.011***		−0.016***	
Constant		2.274***		2.630***		2.810***		2.380***		3.046***		2.847***		1.048***	
Japan: Spline model															
Age categories		Coef	Margins	Coef	Margins	Coef	Margins	Coef	Margins	Coef	Margins	Coef	Margins	Coef	Margins
Pre-pensionable ages	50–59 y	0.014*	—	−0.023***	—	0.007	—	0.002	—	0.032***	—	0.007	—	−0.016***	—
Pensionable ages	60–64 y	−0.003	−0.017	−0.053***	−0.031**	0.003	−0.004	0.021***	0.019	−0.003	−0.035**	0.020***	0.013	−0.024***	−0.008
Post-pensionable ages	65–75 y	−0.005	−0.001	−0.021***	0.032**	0.011	0.008	0.015***	−0.005	0.003	0.006	0.006	−0.015	−0.010***	0.014*
Constant		2.204***	—	2.592***	—	2.815***	—	2.440***	—	2.918***	—	2.860***	—	1.052***	—
LR test: spline versus linear		Spline (*p* = 0.040)		Spline (*p* = 0.009)		Linear (*p* = 0.804)		Linear (*p* = 0.075)		Spline (*p*<0.001)		Linear (*p* = 0.207)		Spline (*p* = 0.037)	
**United States (n = 6198 and 7952)**															
Age specification	Age	Physically demanding		Time pressure		Discretion		Pay		Security		Job satisfaction		Effort reward imbalance	
*n*		6198		6198		6198		6198		6198		7952		6198	
United States: Linear model															
Age	50–75 y	−0.011***		−0.027***		0.013***		0.014***		0.011***		0.014***		−0.013***	
Constant		2.493***		2.367***		3.084***		2.591***		2.951***		3.189***		0.939***	
United States: Spline model															
Age categories		Coef	Margins	Coef	Margins	Coef	Margins	Coef	Margins	Coef	Margins	Coef	Margins	Coef	Margins
Pre-pensionable ages	50–61 y	−0.009*	—	−0.011**	—	0.008*	—	0.009*	—	0.007	—	0.009***	—	−0.004	—
Pensionable ages	62–65 y	−0.015	−0.006	−0.055***	−0.044***	0.036***	0.028**	0.023**	0.014	0.037***	0.030**	0.027***	0.019*	−0.036***	−0.032***
Post-pensionable ages	66–75 y	−0.011	−0.004	−0.031***	0.024	0.002	−0.034**	0.017**	−0.006	−0.006	−0.043**	0.013**	−0.014	−0.011**	0.026**
Constant		2.481***	—	2.282***	—	3.107***	—	2.621***	—	2.966***	—	3.220***	—	0.890***	—
LR test: spline versus linear		Linear (*p* = 0.825)		Spline (*p*<0.001)		Spline (*p* = 0.026)		Linear (*p* = 0.218)		Spline (*p* = 0.008)		Spline (*p* = 0.032)		Spline (*p*<0.001)	

Note: LR test: likelihood ratio test. *p*-values: *** = *p*<0.001, ** = *p*<0.01, * = *p*<0.05. Age centred at 50 years. Age is specified in two ways: a linear specification from 50 to 75 years and as splines in three phases: pre-pensionable ages, pensionable ages and post-pensionable ages. Margins present changes in slope from the previous phase. LR test results indicate whether the spline specification of age provided improved fit.

We tested the second pair of hypotheses – whether attaining pensionable age might mark a break-point in the relationship between job quality and age – by including spline functions with knots delimiting three periods: pre-pensionable ages, pensionable ages and post-pensionable ages. These models were compared to linear specifications with likelihood ratio tests (Table 3). Inclusion of spline functions improved model fit in all three countries for both time pressure and job security and, accordingly, for the composite outcome of effort reward imbalance. Fit was improved by including splines in at least one of the countries for most of the other outcomes, specifically discretion (Sweden and the United States), satisfaction with pay (Sweden) and job satisfaction (Sweden and the United States). Physical labour was better modelled with splines in Japan only in linear random effects modelling, not in the ordinal logistic models sensitivity analyses. Taken as a whole, the findings provide support for hypothesis 2a: *Pensionable ages mark a break-point in the age–job quality relationship*.

To examine how average job quality is after workers reach pensionable age, we display the results as margins in addition to coefficients (Table 3). The margins show whether a slope following a knot has an angle that is different to the angle of the slope before the knot. In general, the age slopes became steeper after the age knot delimiting pensionable and pre-pensionable ages, demonstrating that average job quality is higher after workers reach pensionable age.

**Figure 1.**
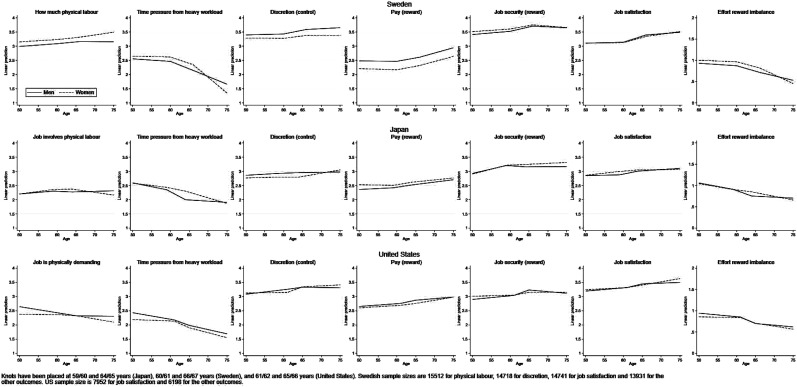
Job quality by age and gender in Sweden (Swedish Longitudinal Occupational Survey of Health, SLOSH, *n* = 13,931 to 15,512), Japan (Japanese Study of Ageing and Retirement, *n* = 3690) and the United States (Health and Retirement Study, *n* = 6198 and 7952).

Turning to the first of the two gender analyses, we found that differences in work quality between men and women were inconsistent across countries (Figure 1 and Table 4). In Sweden, work quality was generally lower for women; compared to men, women reported performing physical labour for a greater part of their working time, greater time pressure, less discretion, lower satisfaction with pay and, correspondingly, greater effort reward imbalance, although women also reported greater job security. Compared to their male compatriots, Japanese women reported greater time pressure and less discretion, but more satisfaction with pay and greater job satisfaction. In the American sample gender differences emerged for certain outcomes, again revealing a mixed picture: while American women reported less physical labour, less time pressure and lower effort reward imbalance than men, they were less satisfied with pay. Support for the hypothesis that there would be gender differences in late career job quality that benefit men was confirmed partially only in the data from Sweden. Instead, the picture was one in which gender differences differed across countries and across outcomes. There was little support for hypothesis 3 that the quality of jobs held in the late career is generally poorer for women than for men.

**Table 4.** Gender interactions in random effects modelling of working conditions and job satisfaction in Sweden (Swedish Longitudinal Occupational Survey of Health), Japan (Japanese Study of Ageing and Retirement) and the United States (Health and Retirement Study).

**Sweden**															
	Work physically (*n* = 15512)		Time pressure from heavy workload (*n* = 13931)		Discretion (*n* = 14718)		Pay (*n* = 13931)		Job security (*n* = 13931)		Job satisfaction (*n* = 14741)		Effort reward Imbalance (*n* = 13931)		
Linear specification	+ Gender	+ Interaction	+ Gender	+ Interaction	+ Gender	+ Interaction	+ Gender	+ Interaction	+ Gender	+ Interaction	+ Gender	+ Interaction	+ Gender		+ Interaction
Age	0.010***	0.010***	—	—	—	—	—	—	—	—	—	—	—		—
Gender (female)	0.152***	0.152***	—	—	—	—	—	—	—	—	—	—	—		—
Gender##Age	—	−0.000	—	—	—	—	—	—	—	—	—	—	—		—
Constant	2.992***	2.992***	—	—	—	—	—	—	—	—	—	—	—		—
Spline specification															
Age 50–60 y	—	—	−0.006***	−0.009***	0.001	0.003	−0.003	−0.001	0.010***	0.011***	0.002	0.003	−0.005***		−0.006***
Age 61–66 y	—	—	−0.049***	−0.052***	0.022***	0.027***	0.024***	0.024***	0.028***	0.031***	0.040***	0.043***	−0.025***		−0.026***
Age 67–75 y	—	—	−0.073***	−0.054***	0.005	0.007	0.036***	0.036**	−0.006	−0.006	0.015***	0.011	−0.028***		−0.021**
Gender (female)	—	—	0.137***	0.095***	−0.151***	−0.110***	−0.287***	−0.266***	0.084***	0.100***	−0.014	0.004	0.086***		0.071***
Gender##Age 50–60 y	—	—	—	0.006	—	−0.004	—	−0.003	—	−0.002	—	−0.002	—		0.002
Gender##Age 61–66 y	—	—	—	0.008	—	−0.010	—	0.001	—	−0.007	—	−0.007	—		0.002
Gender##Age 67–75 y	—	—	—	−0.057**	—	−0.008	—	0.001	—	−0.005	—	0.009	—		−0.020
Constant	—	—	2.530***	2.555***	3.421***	3.397***	2.491***	2.478***	3.421***	3.411***	3.116***	3.105***	0.923***		0.932***
**Japan (*****n***** = 3690)**															
	Physical labour		Time pressure from heavy workload		Discretion		Pay		Job security		Job satisfaction		Effort reward imbalance		
Linear specification	+ Gender	+ Interaction	+ Gender	+ Interaction	+ Gender	+ Interaction	+ Gender	+ Interaction	+ Gender	+ Interaction	+ Gender	+ Interaction	+ Gender	+ Interaction	
Age	—	—	—	—	0.006**	0.005	0.013***	0.015***	—	—	0.011***	0.013***	—	—	
Gender (female)	—	—	—	—	−0.107***	−0.156**	0.100***	0.143**	—	—	0.056**	0.099*	—	—	
Gender##Age	—	—	—	—	—	0.004	—	−0.004	—	—	—	−0.004	—	—	
Constant	—	—	—	—	2.860***	2.881***	2.333***	2.315***	—	—	2.821***	2.802***	—	—	
Spline specification															
Age 50–59 y	0.014*	0.011	−0.022***	−0.027***	—	—	—	—	0.033***	0.031***	—	—	−0.016***		−0.016***
Age 60–64 y	−0.003	−0.006	−0.053***	−0.070***	—	—	—	—	−0.003	−0.009	—	—	−0.024***		−0.032***
Age 65–75 y	−0.005	0.004	−0.020***	−0.009	—	—	—	—	0.003	0.001	—	—	−0.010***		−0.005
Gender (female)	0.033	−0.005	0.121***	−0.003	—	—	—	—	0.045	−0.023	—	—	0.021		−0.014
Gender##Age 50–59 y		0.007	—	0.010	—	—	—	—	—	0.004	—	—	—		0.001
Gender##Age 60–64 y		0.008	—	0.044**	—	—	—	—	—	0.015	—	—	—		0.021**
Gender##Age 65–75 y		−0.023*	—	−0.031**	—	—	—	—	—	0.006	—	—	—		−0.013*
Constant	2.188***	2.205***	2.535***	2.592***	—	—	—	—	2.897***	2.929***	—	—	1.042***		1.059***
**United States**															
	Physically demanding (*n* = 6198)		Time pressure from heavy workload (*n* = 6198)		Discretion (*n* = 6198)		Pay (*n* = 6198)		Job security (*n* = 6198)		Job satisfaction (*n* = 7952)		Effort reward imbalance (*n* = 6198)		
Linear specification	+ Gender	+ Interaction	+ Gender	+ Interaction	+ Gender	+ Interaction	+ Gender	+ Interaction	+ Gender	+ Interaction	+ Gender	+ Interaction	+ Gender	+ Interaction	
Age	−0.012***	−0.016***	—	—	—	—	0.014***	0.015***	—	—	—	—	—	—
Gender (female)	−0.119***	−0.188***	—	—	—	—	−0.064**	−0.053	—	—	—	—	—	—
Gender##Age	—	0.007	—	—	—	—	—	−0.001	—	—	—	—	—	—
Constant	2.565***	2.603***	—	—	—	—	2.628***	2.621***	—	—	—	—	—	—
Spline specification															
Age 50–61 y	—	—	−0.012**	−0.023***	0.008*	0.016**	—	—	0.007	0.013*	0.009***	0.011**	−0.004		−0.009*
Age 62–65 y	—	—	−0.055***	−0.047***	0.036***	0.019	—	—	0.037***	0.047***	0.028***	0.032***	−0.036***		−0.038***
Age 66–75 y	—	—	−0.032***	−0.030***	0.002	−0.002	—	—	−0.005	−0.011	0.013**	0.005	−0.011***		−0.008
Gender (female)	—	—	−0.117***	−0.241***	−0.018	0.048	—	—	0.025	0.120*	0.018	0.047	−0.034**		−0.089**
Gender##Age 50–61 y	—	—	—	0.018*	—	−0.014*	—	—	—	−0.010	—	−0.004	—		0.007
Gender##Age 62–65 y	—	—	—	−0.012	—	0.030	—	—	—	−0.022	—	−0.010	—		0.005
Gender##Age 66–75 y	—	—	—	−0.005	—	0.009	—	—	—	0.012	—	0.017*	—		−0.006
Constant	—	—	2.353***	2.428***	3.119***	3.079***	—	—	2.950***	2.893***	3.211***	3.193***	0.910***		0.944***

Note: Age is centred on 50 years. *p*-values: *** = *p*<0.001, ** = *p*<0.01, * = *p*<0.05.

Turning to the analyses in relation to education level, again differences were inconsistent across countries (Table 5). Support for hypothesis 4, that *the quality of jobs held in the late career is poorer for people with a lower education level compared to people with a higher education level* was provided in Japan and to a degree in the United States. In Sweden, findings were mixed: compared to adults educated up to lower secondary level, more educated older adults reported better job quality in terms of greater discretion, satisfaction with pay, and job security but poorer job quality in terms of spending a greater proportion of the day working physically and greater time pressure. In Japan, compared to people educated up to lower secondary, people with tertiary qualifications reported less physical labour, time pressure and effort reward imbalance, and greater discretion, satisfaction with pay, job security and job satisfaction. Americans with tertiary qualifications reported less physically demanding work, greater satisfaction with pay, higher job security and higher job satisfaction but greater time pressure from a heavy workload than people with lower secondary or fewer qualifications.

**Table 5.** Education interactions in random effects modelling of working conditions and job satisfaction in Sweden (Swedish Longitudinal Occupational Survey of Health), Japan (Japanese Study of Ageing and Retirement) and the United States (Health and Retirement Study).

**Sweden**														
	Work physically (*n* = 15512)		Time pressure from heavy workload (*n* = 13931)		Discretion (*n* = 14718)		Pay (*n* = 13931)		Job security (*n* = 13931)		Job satisfaction (*n* = 14741)		Effort reward imbalance (*n* = 13931)	
Linear specification	+ Education	+ Interaction	+ Education	+ Interaction	+ Education	+ Interaction	+ Education	+ Interaction	+ Education	+ Interaction	+ Education	+ Interaction	+ Education	+ Interaction
Age	0.011***	0.018***	—	—	—	—	—	—	—	—	—	—	—	—
Edu (upper secondary)	0.334***	0.381***	—	—	—	—	—	—	—	—	—	—	—	—
Edu (tertiary)	0.838***	0.944***	—	—	—	—	—	—	—	—	—	—	—	—
Upper sec##Age	—	−0.005	—	—	—	—	—	—	—	—	—	—	—	—
Tertiary##Age	—	−0.013***	—	—	—	—	—	—	—	—	—	—	—	—
Constant	2.603***	2.537***	—	—	—	—	—	—	—	—	—	—	—	—
Spline specification														
Age 50–60 y	—	—	−0.005**	0.012*	0.002	−0.005	−0.002	−0.007	0.010***	0.015***	0.002	−0.001	−0.005***	0.001
Age 61–66 y	—	—	−0.051***	−0.045***	0.022***	0.018**	0.027***	0.017	0.025***	0.034***	0.039***	0.036***	−0.026***	−0.027***
Age 67–75 y	—	—	−0.078***	−0.067*	0.005	−0.004	0.041***	0.086**	−0.009	−0.017	0.015***	0.010	−0.030***	−0.027
Edu (upper secondary)	—	—	0.055**	0.187***	0.059***	−0.015	0.102***	0.056	−0.018	0.031	−0.004	−0.027	0.002	0.044*
Edu (tertiary)	—	—	0.176***	0.342***	0.250***	0.204***	0.145***	0.088*	0.093***	0.145***	0.013	−0.031	0.011	0.062**
Upper sec##Age 50–60 y	—	—	—	−0.016**	—	0.008	—	0.004	—	−0.006	—	0.002	—	−0.005
Tertiary##Age 50–60 y	—	—	—	−0.022***	—	0.006	—	0.007	—	−0.004	—	0.005	—	−0.008**
Upper sec##Age 61–66 y	—	—	—	−0.011	—	0.010	—	0.016	—	−0.003	—	0.006	—	−0.003
Tertiary##Age 61–66 y	—	—	—	−0.004	—	−0.000	—	0.010	—	−0.018*	—	0.003	—	0.005
Upper sec##Age 67–75 y	—	—	—	0.017	—	0.016	—	−0.055	—	0.014	—	0.000	—	0.006
Tertiary##Age 67–75 y	—	—	—	−0.032	—	0.006	—	−0.049	—	0.007	—	0.009	—	−0.010
Constant	—	—	2.511***	2.378***	3.207***	3.262***	2.218***	2.263***	3.441***	3.396***	3.105***	3.134***	0.967***	0.925***
**Japan (*****n***** = 3690)**														
	Physical labour		Time pressure from heavy workload		Discretion		Pay		Job security		Job satisfaction		Effort reward imbalance	
Linear specification	+ Education	+ Interaction	+ Education	+ Interaction	+ Education	+ Interaction	+ Education	+ Interaction	+ Education	+ Interaction	+ Education	+ Interaction	+ Education	+ Interaction
Age	—	—	—	—	0.009***	0.014**	0.016***	0.013**	—	—	0.013***	0.010**	—	—
Edu (upper secondary)	—	—	—	—	0.052	0.174*	0.081**	0.030	—	—	0.019	−0.021	—	—
Edu (tertiary)	—	—	—	—	0.148***	0.171	0.251***	0.174*	—	—	0.158***	0.091	—	—
Upper sec##Age	—	—	—	—	—	−0.009	—	0.004	—	—	—	0.003	—	—
Tertiary##Age	—	—	—	—	—	0.001	—	0.006	—	—	—	0.006	—	—
Constant	—	—	—	—	2.718***	2.646***	2.330***	2.281***	—	—	2.770***	2.811***	—	—
Spline specification														
Age 50–59 y	0.004	0.017	−0.024***	−0.017	—	—	—	—	0.035***	0.024	—	—	−0.017***	−0.012
Age 60–64 y	−0.014	−0.022	−0.055***	−0.031	—	—	—	—	−0.001	−0.003	—	—	−0.025***	−0.011
Age 65–75 y	−0.016**	−0.011	−0.023***	−0.032***	—	—	—	—	0.004	0.005	—	—	−0.012***	−0.017***
Edu (upper secondary)	−0.345***	−0.203	−0.118***	−0.015	—	—	—	—	0.031	−0.077	—	—	−0.066***	0.003
Edu (tertiary)	−0.750***	−0.698***	−0.103*	0.030	—	—	—	—	0.144***	0.046	—	—	−0.114***	−0.015
Upper sec##Age 50–59 y	—	−0.022	—	−0.006	—	—	—	—	—	0.013	—	—	—	−0.003
Tertiary##Age 50–59 y	—	−0.002	—	−0.005	—	—	—	—	—	0.010	—	—	—	−0.008
Upper sec##Age 60–64 y	—	0.020	—	−0.026	—	—	—	—	—	0.007	—	—	—	−0.021*
Tertiary##Age 60–64 y	—	−0.011	—	−0.042	—	—	—	—	—	−0.012	—	—	—	−0.011
Upper sec##Age 65–75 y	—	−0.009	—	0.013	—	—	—	—	—	−0.012	—	—	—	0.011
Tertiary##Age 65–75 y	—	−0.002	—	0.005	—	—	—	—	—	0.040*	—	—	—	−0.005
Constant	2.695***	2.601***	2.696***	2.586***	—	—	—	-	2.838***	2.934***	—	—	1.136***	1.059***
**United States**														
	Physically demanding (*n* = 6198)		Time pressure from heavy workload (*n* = 6198)		Discretion (*n* = 6198)		Pay (*n* = 6198)		Job security (*n* = 6198)		Job satisfaction (*n* = 7952)		Effort reward imbalance (*n* = 6198)	
Linear specification	+ Education	+ Interaction	+ Education	+ Interaction	+ Education	+ Interaction	+ Education	+ Interaction	+ Education	+ Interaction	+ Education	+ Interaction	+ Education	+ Interaction
Age	−0.013***	−0.011	—	—	—	—	0.015***	0.010	—	—	—	—	—	—
Edu (upper secondary)	−0.123*	−0.069	—	—	—	—	0.008	−0.054	—	—	—	—	—	—
Edu (tertiary)	−0.386***	−0.409***	—	—	—	—	0.095*	0.038	—	—	—	—	—	—
Upper sec##Age	—	−0.005	—	—	—	—	—	0.006	—	—	—	—	—	-
Tertiary##Age	—	0.003	—	—	—	—	—	0.005	—	—	—	—	—	—
Constant	2.756***	2.744***	—	—	—	—	2.535***	2.591***	—	—	—	—	—	—
Spline specification														
Age 50–61 y	—	—	−0.011**	0.011	0.008*	0.019	—	—	0.007	0.030	0.009***	−0.014	−0.004	0.007
Age 62–65 y	—	—	−0.053***	−0.050	0.038***	0.008	—	—	0.040***	0.002	0.028***	0.047	−0.036***	−0.050*
Age 66–75 y	—	—	−0.031***	−0.025	0.002	−0.002	—	—	−0.005	−0.000	0.014**	−0.003	−0.011**	−0.006
Edu (upper secondary)	—	—	0.027	0.131	0.100*	0.160	—	—	0.174***	0.358**	0.010	−0.176*	−0.017	0.029
Edu (tertiary)	—	—	0.150**	0.426***	0.193***	0.232*	—	—	0.309***	0.426***	0.085**	−0.106	−0.017	0.090
Upper sec##Age 50–61 y	—	—	—	−0.012	—	−0.013	—	—	—	−0.028	—	0.023*	—	−0.007
Tertiary##Age 50–61 y	—	—	—	−0.033	—	−0.012	—	—	—	−0.021	—	0.025*	—	−0.015
Upper sec##Age 62–65 y	—	—	—	−0.004	—	0.023	—	—	—	0.035	—	−0.021	—	0.013
Tertiary##Age 62–65 y	—	—	—	−0.005	—	0.040	—	—	—	0.046	—	−0.018	—	0.015
Upper sec##Age 66–75 y	—	—	—	−0.001	—	0.010	—	—	—	−0.007	—	0.026	—	−0.003
Tertiary##Age 66–75 y	—	—	—	−0.012	—	0.001	—	—	—	−0.004	—	0.011	—	−0.007
Constant	—	—	2.191***	2.002***	2.962***	2.917***	—	—	2.729***	2.590***	3.171***	3.349***	0.906***	0.830***

Note: Age is centred on 50 years. *p*-values: *** = *p* < 0.001, ** = *p* < 0.01, * = *p* < 0.05.

We next examined whether there was effect modification by gender or education level in the job quality–age relationship. For gender, results from stratified analyses are presented in Figure 1 and interactions are reported in Table 4. Interaction terms were seldom significant at the 5% level; exceptions were the time pressure–age relationship which varied by gender but inconsistently across countries, and a small but significant difference for discretion (women aged 50–61 years) in the United States and in the ordered logistic regression sensitivity analysis only for Sweden. In short, we did not observe differences by gender in the development of working conditions by age and could not confirm hypothesis 5: *the effects of attaining pensionable age on the age–job quality relationship are smaller for women than for men*. For education level, results from interactions are reported in Table 5. Very few interactions were significant: consistent findings in both the linear and ordered logistic models were, from Sweden at ages 50–60, an increase in time pressure for those with up to lower secondary education and decreases in time pressure in the more educated groups; in the United States, increases in job satisfaction in 50–61 year olds with more than secondary education that were observed only in the linear models. Almost no interactions were significant in any of the countries in the older age groups. We could not find support for hypothesis 6 that *the effects of attaining pensionable age on the age–job quality relationship are smaller for people with a lower education level compared to people with a higher education level*.

We lastly examined whether age differences in job quality were more distinct in Sweden than in Japan or the United States, per hypothesis 7: *The effects of attaining pensionable age on the age–job quality relationship may be larger in Sweden than in the United States or Japan*. In the linear specification, there was no indication of stronger age–job quality relationships in Sweden than in the other two countries. The most prominent differences among the countries concerned the measure of physical working conditions, where increasing age was associated with reporting more time working entirely physically in Sweden, no discernible difference in Japan and with less physically demanding work in the United States, a finding that ran counter to our expectations. Spline functions were more often the better fitting model compared with linear functions in Sweden and the United States than in Japan, but there was no indication that the gradients at pensionable ages were systematically steeper in Sweden than in the United States. In short, there was little support for the hypothesis that age differences in psychosocial job quality and job satisfaction were more distinct in Sweden than in the other two countries.

## Discussion

This paper uses panel data from Japan, Sweden and the United States to find that job quality was consistently better among people working after pensionable age than for those still in their fifties. These findings were observed across a range of psychosocial work exposures as well as job satisfaction, bolstering limited existing evidence and extending knowledge to countries outside Europe (Åkerstedt et al., 2019; Brussig, 2016; Dingemans & Henkens, 2020; Wahrendorf et al., 2017). Interestingly, these findings run counter to the reasonable expectation that factors such as financial pressures and ageism would lead to downgrading of job quality in later life (Lain, 2012); on the contrary, even factors like job security and satisfaction with pay were rated more positively among participants older than pensionable age compared to participants in their fifties. The findings from spline modelling suggest that differences in job quality can be linked to pensionable ages, suggesting a role for the protection provided by pensions in promoting better working conditions among older workers. Financial security provided by old-age pensions may provide older workers with leverage that lets them refuse poor quality work and craft jobs that suit them better. These processes may drive up job quality (Platts et al., 2021), potentially enabling older workers to remain in paid work longer than they otherwise might have.

This study also focused on potential social and gender inequalities in access to high quality jobs in later life. We found that only in Sweden did women tend to have poorer quality (if more secure) jobs than men, this finding being consistent with Damman and Henkens (2020). Older workers with more formal qualifications tended to report more discretion, greater satisfaction with pay and more job security. We did not, however, find evidence for gender or education differences in the relationship between job quality and age. One interpretation for this finding – that any gender or education differences tended to be observed in the main effects rather than in the interactions with age – is that earlier-career inequalities persist and extend into post-pensionable ages. Further, our findings imply that women, by being more likely to retire early, may more often miss out on the experience of high quality paid work later in life (Wahrendorf et al., 2017).

Despite differences between Sweden, Japan and the United States in labour participation rates, poverty rates and pensions generosity, the overall impression is one of similarities between the countries. Relationships between age and job quality did not emerge from national particularities, suggesting that jobs held after pensionable age may generally be high quality in countries with mature welfare states. The role of pension eligibility could be tested by extending study of working conditions in these age groups to lower and middle income countries with incomplete old-age pension coverage (Lloyd-Sherlock, 2010). In such contexts, there may be pressure to supplement any pension income with labour earnings, even in poor quality jobs, and rising job quality after pensionable age might not be observed. Such a comparison would provide stronger evidence for any role of old-age pensions in raising job quality in the late career.

### Conceptualizing the Late Career in Two Phases: Perspectives for Future Research

The evidence presented in this paper has implications for how the late career is conceptualized. The late career is typically viewed as a single period extending to retirement from about age 55. We observed that working conditions of post-pensionable-age jobs are distinctive, evidence which support a two-phase division of the late career. The first phase – commencing in the fifties and extending up to pensionable age – has been much-studied and is generally characterized by diminished labour market opportunities. The second phase – following age of eligibility for old-age pensions – is characterized by reduced financial and normative pressures to work (Platts et al., 2021). This phase is shaped by taken-for-granted cultural scripts and age-graded institutional arrangements (e.g. old-age pensions, health insurance, mandatory retirement) encouraging withdrawal from the labour market (Moen, 2011). Consequently, if older adults are in paid work, it is more likely to be freely chosen and fit individuals' goals and preferences (Moen, 2007; Moen & Chermack, 2005). The current study contributes to growing evidence that adults over pensionable age are strategically seeking and finding paid work that is less stressful, freer and more rewarding (Platts et al., 2021; Sacco et al., 2021).

This perspective opens up several avenues for further investigation, the first being the likelihood of experiencing better working conditions in the latter part of the late career for any given individual. How likely is it that a person in paid work at age 55 will remain in work and experience better working conditions in their late sixties? There may be substantial differences in relation to societal context and individual characteristics, be they ascribed characteristics such as race and gender or earlier circumstances and events (Krekula et al., 2017; McAllister et al., 2019). Future research could incorporate trajectories involving withdrawal from the labour market while measuring temporal variations in working conditions.

A second avenue concerns exploration of mechanisms driving better working conditions in the latter part of the late career and their implications for inequalities. It was not the focus of this paper to establish how far higher job quality in later life resulted from selection of those with poorer quality jobs out of the in-work sample or from improvements to job quality participants experienced. If the sole mechanism responsible is that workers with poorer working conditions are differentially selected into retirement once they are eligible for old-age pensions (Carr et al., 2016; Smeaton et al., 2009), such processes may deepen financial inequalities in later life. The other mechanism – workers experiencing improvements in working conditions as they age, which has been observed in Sweden (Sacco et al., 2021) – would impact inequalities to a lesser degree. Future research could explore which mechanism tends to dominate in different groups and national settings.

A third avenue involves exploring older workers’ strategies for exercising agency. In this ‘do-it-yourself’ life phase, individuals are required to reflexively manage their working lives (Moen, 2011) and may be able to craft their jobs to make tasks and relationships more amenable (Kooij et al., 2015; Wong & Tetrick, 2017; Wrzesniewski & Dutton, 2001). Evidence from Europe and the United States suggests that older workers experienced improved psychosocial working conditions after switching into bridge jobs or reducing their working hours (Johnson et al., 2009; Nekola et al., 2018; Platts et al., 2021; Sacco et al., 2021). Future research could assess whether job changes or reductions in working hours account for some of the differences observed in the current study (Sacco et al., 2021).

A fourth avenue concerns the nature of trade-offs in work quality characteristics that are taking place. While we observed perceptions of job quality that improved across the board, this study included a subset of work quality characteristics, and did not capture the full financial gains, social status, and array of nonpecuniary costs and benefits that come with paid work. Future work could test more thoroughly the arguments presented here that workers experience improvements in overall compensation from their jobs later in life, as opposed to making trade-offs that lead to both gains and losses in job quality.

### Limitations

This study uses large panels harmonized *ex post* to compare a range of job quality measures for three countries characterized by late labour force exits. First, the sample sizes vary across the countries, which may have affected the degree to which it was possible to observe spline relationships, particularly in Japan where the findings for pay satisfaction approached statistical significance. Second, the outcome measures for this study are based on individuals’ subjective perceptions of working conditions, as opposed to objective measures, and might not necessarily correspond with actual improvements. Further, such perceptions may be affected differentially by gender and social position. Potentially, workers may be exhibiting a positivity bias with age (Carstensen, 2006). However, the age differences we observed were specific, suggesting they are to do with actual changes in the work environment: reductions in time pressure due to a heavy workload were larger than the increases observed in the global measure, most susceptible to positive bias, of job satisfaction. Third, we were able to include some study of individual differences by incorporating gender and education level. While we did not find evidence of heterogeneity in the associations between age and job quality in terms of these characteristics, this does not exclude the possibility of future work identifying areas of the labour market where age-related declines in working conditions dominate. Investigating other aspects of individual heterogeneity, such as social status, financial insecurity and health, and specific vulnerabilities relating to their intersections (de los Reyes, 2017), would be promising avenues for future research. Fourth, participants with poorer working conditions may have been less likely than others to participate in follow-up waves, limiting the generalizability of the findings to such groups. Lastly, while we have presented the findings as linear and non-linear age effects, this interpretation is based on assumptions of negligible period and cohort effects. Empirically, inclusion of period effects in sensitivity analyses did little to change the findings (cf. Supplementary Table A2 in appendix). Although it is difficult to identify whether linear processes are caused by age, period or cohort, non-linear trends can reliably point to ruptures, such as those we observe in relation to pensionable ages (Bell, 2020; Glenn, 2005).

## Conclusion

This study uses data from Sweden, Japan and the United States to find that job quality is better after pensionable age. Although the late career is typically viewed as a single period starting around age 55, we argue for importance of distinguishing a second late career phase that begins at pensionable age. At a life phase when norms and institutions encourage retirement lifestyles, study of this little-researched career phase offers insights into the strategies and trade-offs that older adults make, the constraints that they face, and processes leading to inequalities in later life.

## Supplemental Material

sj-pdf-1-roa-10.1177_01640275221075985 – Supplemental Material for Job Quality in the Late Career in Sweden, Japan and the United States Click here for additional data file. Supplemental Material, sj-pdf-1-roa-10.1177_01640275221075985 for Job Quality in the Late Career in Sweden, Japan and the United States by Loretta G. Platts, Lawrence B. Sacco, Ayako Hiyoshi, Hugo Westerlund, Kevin E. Cahill and Stefanie König in Research on Aging
