# Supplementary material for: Job Quality in the Late Career in Sweden, Japan and the United States
Source: Res Aging. 2022 May 19;45(3-4):259–79. doi: 10.1177/01640275221075985 (PMC10021131; doi:10.1177/01640275221075985)
Supplement: sj-pdf-1-roa-10.1177_01640275221075985 – Supplemental Material for Job Quality in the Late Career in Sweden, Japan and the United States [file sj-pdf-1-roa-10.1177_01640275221075985.pdf]

Job quality in the late career in Sweden, Japan and the United States

Appendix

Table A1: Harmonized variables in the Swedish Longitudinal Occupational Survey of Health, Japanese Study of Ageing and Retirement and the US Health and Retirement Study

Table A2: Random effects modelling of working conditions and job satisfaction after inclusion of linear functions for period in Sweden (Swedish Longitudinal Occupational Survey of Health), Japan (Japanese Study of Ageing and Retirement) and the United States (Health and Retirement Study)

Figure A1: Sample selection in the Swedish Longitudinal Occupational Survey of Health

Table A1: Harmonized variables in the Swedish Longitudinal Occupational Survey of Health, Japanese Study of Ageing and Retirement and the United States Health and Retirement Study

**Concept**	**Swedish Longitudinal Occupational Survey of Health**	**Japanese Study of Ageing and Retirement**	**United States Health and Retirement Study**
Demographics			
Gender	Obtained from the longitudinal integrated database for health insurance and labour market studies (LISA) register.	Self-reported.	Self-reported.
Age	Obtained from the longitudinal integrated database for health insurance and labour market studies (LISA) register.	Self-reported.	Self-reported.
Job quality			
Physical work (efforts)	*Worker questionnaire waves 2–7.* At certain times does your job involve working entirely physically, in other words doing more with your body than one does when one goes and stands and moves in a usual way? 1=Nearly all the time, 2=About three-quarters of the time, 3=Half of the time, 4=About one quarter of the time, 5=A little, perhaps one-tenth of the time, 6=No, not at all.	"My current job requires physical labour." To what extent do you agree or disagree with the statement? 1=Strongly agree, 2=Moderately agree, 3=Moderately disagree, 4=Strongly disagree, 5=Don't know, 6=Refused to answer.	*Waves 8–11 at alternate waves.* "My job is physically demanding." 1=Strongly disagree, 2=Disagree, 3=Agree, 4=Strongly agree, 5=Does not apply.
Time pressure due to heavy workload (efforts)	*Worker questionnaire waves 3–7.* "Due to a heavy workload I often work under great time pressure." 1=This is completely correct, 2=This is quite correct, 3=This is not really correct, 4=This is not correct at all.	"I have so much work to do that I always feel pressed for time." To what extent do you agree or disagree with the statement? 1=Strongly agree, 2=Moderately agree, 3=Moderately disagree, 4=Strongly disagree, 5=Don't know, 6=Refused to answer.	*Waves 8–11 at alternate waves.* "I am under constant time pressure due to a heavy workload." 1=Strongly disagree, 2=Disagree, 3=Agree, 4=Strongly agree, 5=Does not apply.
Discretion (control)	*Worker questionnaire waves 3–7, non-worker questionnaire waves 5–7.* Do you have a choice in deciding how you do your work? 1=Yes, often, 2=Yes, sometimes, 3=No, rarely, 4=No, never, as good as never.	"At my current job, I have little discretion over how to proceed with my tasks. In other words, I can't decide about anything on my own." To what extent do you agree or disagree with the statement? 1=Strongly agree, 2=Moderately	*Waves 8–11 at alternate waves.* "I have very little freedom to decide how I do my work." 1=Strongly disagree, 2=Disagree, 3=Agree, 4=Strongly agree, 5=Does not apply.

		agree, 3=Moderately disagree, 4=Strongly disagree, 5=Don’t know, 6=Refused to answer.	
Satisfaction with pay (reward)	*Worker questionnaire waves 3–7.*<br>“Considering all my efforts and achievements, my pay is adequate.” 1=This is completely correct, 2=This is quite correct, 3=This is not really correct, 4=This is not correct at all.	“Taking all factors into consideration, including my efforts and dedication as well as the business performance of the company, I am satisfied with my current pay.” To what extent do you agree or disagree with the statement? 1=Strongly agree, 2=Moderately agree, 3=Moderately disagree, 4=Strongly disagree, 5=Don’t know, 6=Refused to answer.	*Waves 8–11 at alternate waves.*<br>“My salary is adequate.” 1=Strongly disagree, 2=Disagree, 3=Agree, 4=Strongly agree, 5=Does not apply.
Job security (reward)	*Worker questionnaire waves 3–7.*<br>“My job security is poor.” 1=This is completely correct, 2=This is quite correct, 3=This is not really correct, 4=This is not correct at all.	What do you think is the likelihood of losing your current job for a reason other than reaching your retirement age? 1=Very likely, 2=Fairly likely, 3=Fairly unlikely, 4=Very unlikely, 5=Don’t know, 6=Refused to answer.	*Waves 8–11 at alternate waves.*<br>“My job security is poor.” 1=Strongly disagree, 2=Disagree, 3=Agree, 4=Strongly agree, 5=Does not apply.
Job satisfaction	*Worker questionnaire waves 3–7, non-worker questionnaire waves 4–7.*<br>All in all, are you satisfied with your work? Polar-labelled 1–8 scale, where 1=Very dissatisfied” and 8=Very satisfied.	“Overall, I am satisfied with my current job.” To what extent do you agree or disagree with the statement? 1=Strongly disagree, 2=Moderately disagree, 3=Moderately agree, 4=Strongly agree, 5=Don’t know, 6=Refused to answer.	*Waves 8–13 at alternate waves.*<br>“All things considered, I am satisfied with my job.”<br>1=Strongly disagree, 2=Disagree, 3=Agree, 4=Strongly agree, 5=Does not apply.
Health			
Self-rated health	*Worker questionnaire waves 1–7, non-worker questionnaire waves 2–7.*<br>How do you evaluate your state of health? 1=Very good, 2=Quite good, 3=Neither good nor bad, 4=Quite bad, 5=Very bad.	Which of the following statements most accurately describes the current status of your overall health condition? 1=Very good, 2=Good, 3=Relatively good, 4=Poor, 5=Very poor, 6=Don’t know.	*All waves.*<br>Would that you say your health is excellent, very good, good, fair, or poor? 1=Excellent, 2=Very good, 3=Good, 4=Fair, 5=Poor.

Note: The table lists original questionnaire items and responses in English translation where necessary. Responses are presented before any rescaling and/or reverse-scaling was performed by the authors.

Table A2: Random effects modelling of working conditions and job satisfaction after inclusion of linear functions for period in Sweden (Swedish Longitudinal Occupational Survey of Health), Japan (Japanese Study of Ageing and Retirement) and the United States (Health and Retirement Study)

**Sweden with wave controls (n=13931 to 15512)**															
	Age	Work physically		Time pressure		Discretion		Pay		Security		Job satisfaction		Effort reward imbalance	
n		15512		13931		14718		13931		13931		14741		13931	
Sweden: Linear model															
Age	50–75 y	0.004**		−0.024***		0.009***		0.003*		0.011***		0.015***		−0.011***	
Wave		0.017***		0.032***		−0.010***		0.025***		0.033***		−0.005		−0.001	
Constant		3.038***		2.526***		3.347***		2.187***		3.300***		3.078***		1.004***	
Sweden: Spline model															
		Coef.	Margins	Coef.	Margins	Coef.	Margins	Coef.	Margins	Coef.	Margins	Coef.	Margins	Coef.	Margins
Wave		0.017***	-	0.032***	-	−0.010***	-	0.025***	-	0.033***	-	−0.005*	-	−0.001	-
Pre-pensionable ages	50–60 y	0.003*	-	−0.010***	-	0.002	-	−0.006***	-	0.008***	-	0.003*	-	−0.004***	-
Pensionable ages	61–66 y	0.007*	0.004	−0.054***	−0.044***	0.024***	0.022***	0.024***	0.030***	0.024***	0.017***	0.040***	0.037***	−0.025***	−0.021***
Post-pensionable ages	67–75 y	−0.003	−0.010	−0.084***	−0.030**	0.009	−0.015*	0.036***	0.012	−0.015	−0.040***	0.016***	−0.024***	−0.029***	−0.004
Constant		3.040***	-	2.467***	-	3.377***	-	2.224***	-	3.312***	-	3.131***	-	0.978***	-
LR test: spline vs linear		Linear (p=0.406)		Spline (p<0.001)		Spline (p<0.001)		Spline (p<0.001)		Spline (p<0.001)		Spline (p<0.001)		Spline (p<0.001)	
**Japan with wave controls (n=3690)**															
	Age	Physical labour		Time pressure		Discretion		Pay		Security		Job satisfaction		Effort reward imbalance	
Japan: Linear model															
Age	50–75 y	0.001		−0.032***		0.006**		0.010***		0.009***		0.011***		−0.015***	
Wave		0.009		−0.001		0.014		0.042***		0.018		−0.000		−0.012*	
Constant		2.264***		2.632***		2.785***		2.315***		3.014***		2.847***		1.067***	
Japan: Spline model															
		Coef.	Margins	Coef.	Margins	Coef.	Margins	Coef.	Margins	Coef.	Margins	Coef.	Margins	Coef.	Margins
Wave		0.006	-	−0.000	-	0.015	-	0.045***	-	0.014	-	−0.001	-	−0.011*	-
Pre-pensionable ages	50–59 y	0.013*	-	−0.023***	-	0.005	-	−0.004	-	0.031***	-	0.008	-	−0.014***	-
Pensionable ages	60–64 y	−0.004	−0.017	−0.053***	−0.031**	0.002	−0.004	0.017*	0.020*	−0.004	−0.035**	0.020***	0.013	−0.023***	−0.008
Post-pensionable ages	65–75 y	−0.005	−0.001	−0.021***	0.032**	0.010	0.009	0.014**	−0.003	0.002	0.006	0.006	−0.015	−0.009***	0.013*
Constant		2.199***	-	2.593***	-	2.792***	-	2.385***	-	2.898***	-	2.861***	-	1.067***	-
LR test: spline vs linear		Linear (p=0.051)		Spline (p=0.009)		Linear (p=0.738)		Spline (p=0.019)		Spline (p<0.001)		Linear (p=0.206)		Linear (p=0.060)	

United States with wave controls (n=6198 and 7952)															
Age specification	Age	Physically demanding		Time pressure		Discretion		Pay		Security		Job satisfaction		Effort reward imbalance	
n		6198		6198		6198		6198		6198		7952		6198	
United States: Linear model															
Age	50–75 y	−0.012***		−0.027***		0.013***		0.014***		0.010***		0.014***		−0.013***	
Wave		0.041***		−0.052***		−0.051***		−0.003		−0.061		0.010**		0.036***	
Constant		2.399***		2.240***		3.211***		2.598***		3.101***		3.157***		0.852***	
United States: Spline model															
		Coef.	Margins	Coef.	Margins	Coef.	Margins	Coef.	Margins	Coef.	Margins	Coef.	Margins	Coef.	Margins
Wave		−0.041***	-	−0.050***	-	−0.050***	-	−0.002	-	−0.059***	-	0.010**	-	0.035***	-
Pre-pensionable ages	50–61 y	−0.011**	-	−0.012**	-	0.009*	-	0.009*	-	0.008*	-	0.008**	-	−0.005*	-
Pensionable ages	62–65 y	−0.011	−0.000	−0.050***	−0.038**	0.030***	0.022*	0.023*	0.014	0.031***	0.023*	0.028***	0.020*	−0.033***	−0.028***
Post-pensionable ages	66–75 y	−0.014*	−0.002	−0.033***	0.017	0.004	−0.026*	0.017**	−0.006	−0.003	−0.034*	0.013**	−0.015	−0.012***	0.020*
Constant		2.394***	-	2.165***	-	3.226***	-	2.625***	-	3.105***	-	3.188***	-	0.811***	-
LR test: spline vs linear		Linear (p=0.934)		Spline (p<0.001)		Linear (p=0.108)		Linear (p=0.225)		Spline (p=0.047)		Spline (p=0.019)		Spline (p<0.001)	

Note: LR test: likelihood ratio test. P-values: ***=p<0.001, **=p<0.01, *=p<0.05. Age centred at 50 years. Age is specified in two ways: a linear specification from 50–75 years and as splines in three phases: pre-pensionable ages, pensionable ages and post-pensionable ages. Margins present changes in slope from the previous phase. LR test results indicate whether the spline specification of age provided improved fit.

Figure A1: Sample selection for the Swedish Longitudinal Occupational Survey of Health

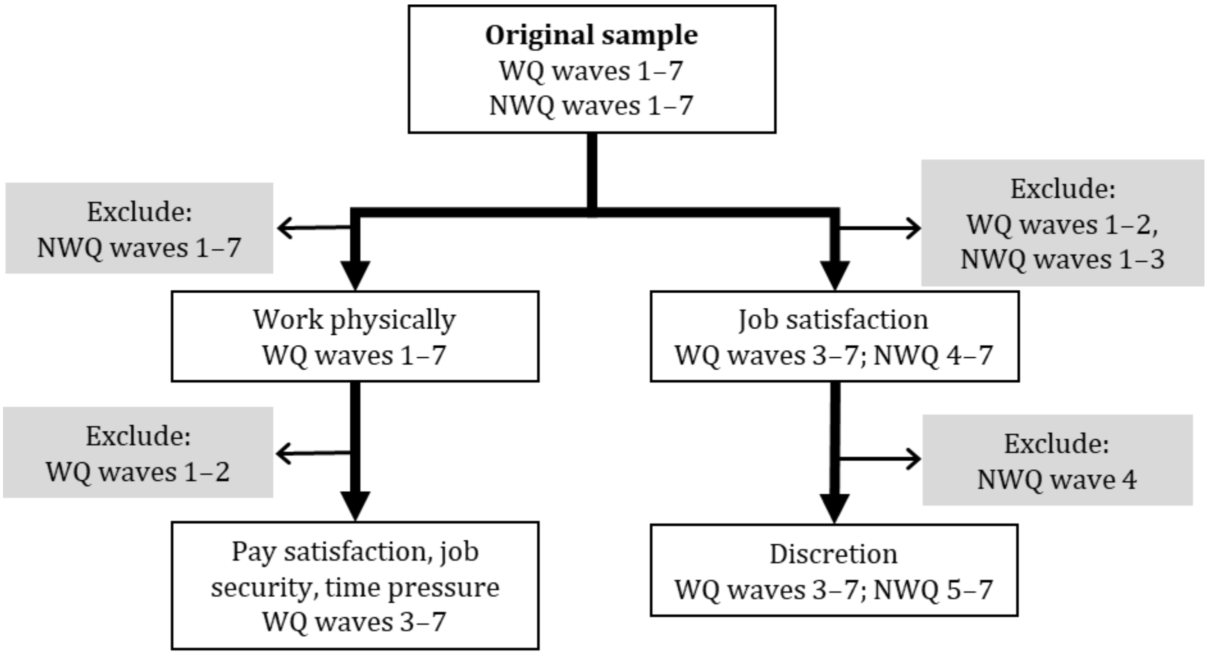

Note: WQ: Questionnaire for participants in paid work ≥30% of full-time; NWQ: Questionnaire for participants who worked <30% or did not work.
